# Association of dietary patterns with hypertension among adults residing in Tibetan China: findings from a population-based study

**DOI:** 10.3389/fnut.2025.1534915

**Published:** 2025-03-13

**Authors:** Xinran Li, Xin Zhang, Qiling Gou, Qingtao Meng, Xiaoping Chen

**Affiliations:** ^1^Department of Cardiology, West China Hospital, Sichuan University, Chengdu, Sichuan, China; ^2^Department of Cardiovascular Medicine, Shaanxi Provincial People's Hospital, Xi'an, Shaanxi, China

**Keywords:** Tibetan, dietary patterns, hypertension, food frequency questionnaire, principal component analysis

## Abstract

**Objectives:**

This study aimed to investigate the dietary patterns of Tibetan residents and explore their association with the prevalence of hypertension.

**Methods:**

A multi-stage, stratified, random sampling method was employed to include Tibetan residents from Luhuo County, Garze Tibetan Autonomous Prefecture, Sichuan Province, China. Dietary information was collected through face-to-face interviews using a Food Frequency Questionnaire (FFQ) consisting of 92 food items. Participants were asked to report the frequency and portion size of their consumption of each food item over the past year. The collected data were subsequently converted into average daily intake, with the 92 food items grouped into 23 distinct categories. Principal Component Analysis (PCA) was then used to identify dietary patterns. Binary logistic regression analysis was conducted to investigate the association between dietary patterns and the prevalence of hypertension, adjusting for potential confounders including age, gender, living area, education, physical activity, current smoking, current alcohol consumption, diabetes, dyslipidemia, and overweight/obesity. A *P* value <0.05 was considered statistically significant.

**Results:**

A total of 1,262 Tibetan residents participated in the study, with an average age of 46 ± 15 years. Among them, 36.8% were male, and the prevalence of hypertension was 30.2%. Three distinct dietary patterns were identified among Tibetan residents and were subsequently named as the “Tsamba-red meat-tuber,” “Rice-vegetable-fruit,” and “Dairy products” patterns. After adjusting for confounding factors, individuals in the highest quartile following the “Tsamba-red meat-tuber” pattern were found to be associated with a higher prevalence of hypertension (OR = 3.04, 95% CI: 2.06–4.50; *P* for trend <0.001). In contrast, individuals in the highest quartile following the “Rice-vegetable-fruit” pattern were associated with a lower prevalence of hypertension (OR = 0.45, 95% CI: 0.30–0.67; *P* for trend <0.001). Additionally, those in the highest quartile of the “Dairy products” pattern also showed a lower prevalence of hypertension (OR = 0.58, 95% CI: 0.39–0.85; *P* for trend = 0.002).

**Conclusion:**

The “Tsamba-red meat-tuber” pattern is associated with a higher risk of hypertension, whereas the “Rice-vegetable-fruit” and “Dairy products” patterns are associated with a lower risk of hypertension in this population.

## 1 Introduction

Hypertension is a major risk factor for cardiovascular and cerebrovascular diseases (1). Among the primary ethnic groups in China, Tibetans, numbering over 6 million, have been shown to exhibit a high prevalence of hypertension, ranging from 23.4 to 55.9% (2–4), with this heterogeneity in prevalence potentially attributable to differences in altitude (5) and lifestyle factors (2, 6) among the studied populations. These rates are substantially higher than the national average of 23.5% (7). Hypertension is influenced by both genetic and environmental factors, with diet being one of the most significant environmental contributors (8). The high-altitude, low-oxygen environment of Tibetan regions has shaped unique dietary habits, including a preference for tsamba, beef and mutton, and dairy products. However, the detailed characteristics of the Tibetan diet and its relationship with hypertension remain inadequately understood.

To comprehensively evaluate the relationship between diet and disease, nutritionists have proposed the concept of dietary patterns (9). Dietary patterns take into account multiple foods and nutrients as a whole, emphasizing their interactions, and thus better reflect the impact of overall dietary exposure on disease (10, 11). Research evidence suggests that dietary patterns, such as the Mediterranean diet and the Dietary Approaches to Stop Hypertension (DASH) diet, are strongly associated with a lower risk of hypertension, while the Western diet is correlated with a higher risk (12). A longitudinal study from the China Health and Nutrition Survey (1991–2018) found that adherence to the modern dietary pattern, characterized by a high intake of fruits and dairy products, was negatively associated with systolic blood pressure (SBP), while the meat-based dietary pattern was positively associated with diastolic BP (DBP) and the risk of hypertension (13). However, few studies have investigated the link between Tibetan dietary patterns and hypertension. A cross-sectional study conducted in Diqing, Yunnan, which included a multi-ethnic population with 35% Tibetans, identified three major dietary patterns: ‘Grassland healthy,’ ‘Tuber and meat,’ and ‘Fruit and vegetable.’ The ‘Grassland healthy’ pattern was found to be associated with a lower risk of hypertension (14). Additionally, a cohort study of Tibetan adults in the Tibetan Plateau region identified three primary dietary patterns: modern, urban, and pastoral dietary patterns. The modern dietary pattern was positively associated with elevated BP, while the pastoral dietary pattern showed a negative association with elevated BP (15). Tibetans are mainly distributed across southwestern China, including Tibet, Sichuan, Qinghai, Yunnan, and other regions. Garze Tibetan Autonomous Prefecture in Sichuan is the second-largest Tibetan area in China. This region not only preserves traditional Tibetan dietary habits but also absorbs influences from the culinary culture of southwestern China. Furthermore, with improved transportation access, the diversity of available ingredients has significantly increased. As a result, Garze Tibetan Autonomous Prefecture has developed a unique dietary profile. However, no studies have yet explored the dietary patterns in this region or their association with hypertension.

Therefore, this study aims to investigate the dietary patterns of Tibetan residents in Ganzi Tibetan Autonomous Prefecture using a food frequency questionnaire (FFQ) and explore their potential correlation with hypertension risk.

## 2 Materials and methods

### 2.1 Subjects

**Figure 1:**
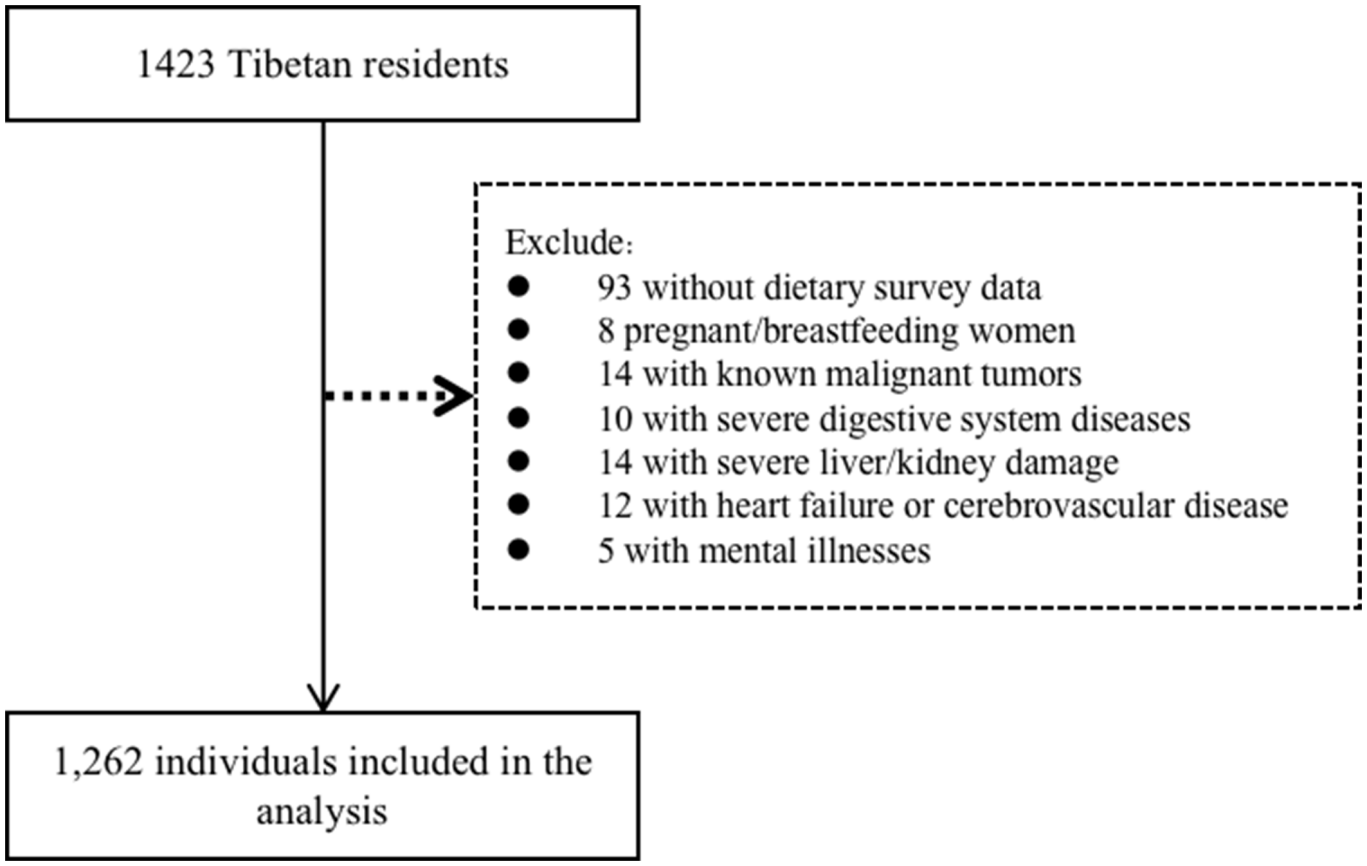
Flow diagram illustrating the study population inclusion process.

Luhuo County is located in the central and northern parts of the Garze Tibetan Autonomous Prefecture in Sichuan Province and is a semi-agricultural, semi-pastoral area, with the majority of the population being Tibetan (94.8%). The locals have preserved traditional Tibetan food habits and ways of life. Therefore, we conducted a multi-stage, stratified, and randomly sampled survey in Luhuo County from January 2018 to October 2020. First, two towns were selected from each of the four administrative districts in Luhuo County. Then, 2–3 villages were selected from each town, and finally, 95 Tibetan residents were randomly selected in each village, all of whom were aged between 18 and 80 years. We excluded the following individuals from the study: those with self-reported mental illnesses, serious physical illnesses, or visual/hearing impairments; those with a recent history of angina, acute myocardial infarction, heart failure, or cerebrovascular disease within the past 6 months; those with severe liver/kidney damage (serum alanine aminotransferase or aspartate aminotransferase >2 times the upper limit of normal, serum creatinine >260 μmol/L); those with severe digestive system diseases or known malignant tumors; and pregnant or breastfeeding women. A total of 1,262 subjects, with data on dietary surveys and medical history, were included in the study, out of 1,423 participants overall (Figure 1). This study was approved by the Ethics Committee of West China Hospital, Sichuan University. All participants provided informed consent after being informed of the objectives and potential benefits of the study.

### 2.2 Dietary assessment

According to our prior dietary survey of 86 Tibetan residents, we compiled a list of foods commonly consumed by this population. Using nutritional epidemiology methods as a reference (16), we developed a Food Frequency Questionnaire (FFQ) containing 92 food items for dietary assessment. Our trained investigators, with the assistance of local Tibetan translators, conducted face-to-face dietary surveys with the participants. Participants were asked to report the frequency and portion size of each food item consumed over the past year. Standard containers, such as bowls, cups, and spoons with marked scales, as well as concentric circles of different sizes and pictures of foods, were used to assist participants in recalling their food intake. The portion weights for each food item were determined in advance. The dietary information obtained from the FFQ was subsequently converted into average daily intake, calculated by multiplying the intake frequency by the portion size and its corresponding weight. To facilitate the identification and interpretation of dietary patterns in subsequent analyses, we utilized the classification of the China Food Composition Table (17) and considered the dietary habits of the Tibetan region. The 92 food items listed in the FFQ were consolidated into 23 distinct food groups, including rice and its products, wheat and its products, tsamba, whole grains, tubers, beans and their products, vegetables, mushrooms, pork, beef and mutton, poultry, animal organs, aquatic products, eggs, dairy and its products, fruits, nuts, pastries, butter tea/mike tea, sweet beverages, tea, salt, and oils. Among these, tsamba, a staple food crop widely consumed by Tibetans, is made from roasted Tibetan barley (13, 18).

### 2.3 Covariates

#### 2.3.1 Covariates measurement and collection

The interview involved the collection of demographic data (e.g., sex, education, living area), clinical data (e.g., current smoking status, medical history), and information related to lifestyle (e.g., physical activity).

Participants’ height and weight were measured while they were wearing lightweight clothing and without shoes, with precision to 1 cm and 0.2 kg. Body mass index (BMI) was calculated as weight divided by height squared (kg/m^2^).

BP was measured using a standardized automatic electronic sphygmomanometer (Omron HBP-1100), following a standardized protocol (19). Readings were taken three times at 2-min intervals after at least 5 min of rest in a warm and quiet indoor setting. The mean value of the three readings was used in subsequent statistical analyses.

Blood samples were drawn from the antecubital vein in the morning after an 8-h fast. The blood samples were then transported to the Department of Laboratory Medicine at West China Hospital of Sichuan University and analyzed for fasting blood glucose (FBG), fasting serum total cholesterol (TC), low-density lipoprotein cholesterol (LDL-C), high-density lipoprotein cholesterol (HDL-C), and triglycerides (TG) using an automatic biochemistry analyzer (Roche Cobas8000).

#### 2.3.2 Related definitions of covariates

Hypertension was defined as a SBP of at least 140 mmHg and/or a DBP of at least 90 mmHg, or self-reported current use of antihypertensive medications (20). Diabetes was defined as a fasting blood glucose level of at least 7.0 mmol/L, or self-reported current use of insulin or hypoglycemic agents (21). Dyslipidemia was defined as the self-reported current use of antilipidemic medications or meeting at least one of the following criteria: TC ≥ 5.2 mmol/L, LDL-C ≥ 3.4 mmol/L, HDL-C < 1.0 mmol/L, or TG ≥ 1.7 mmol/L (22). BMI was categorized as overweight (BMI 24–27.9 kg/m^2^) and obesity (BMI ≥ 28 kg/m^2^). Current smoking was defined as smoking at least one cigarette per day within the past year, while current drinking was defined as consuming alcohol at least once per week within the past year. Physical activity levels were classified into four categories based on daily activity duration: <30 min (level 1), 30 min to 1 h (level 2), 1 to 1.5 h (level 3), and > 1.5 h (level 4) (19).

### 2.4 Statistical analyses

Dietary patterns were identified using Principal Component Analysis (PCA). Prior to conducting the analysis, the Kaiser-Meyer-Olkin measure of sample and Bartlett test of sphericity were employed to evaluate the suitability of the data for factor analysis (23). A total of 23 factors were initially extracted. Based on eigenvalues (>1.0), the scree plot (indicating the point where the slope levels off), the proportion of variance explained by each factor (>5%), and the interpretability of the factors, three primary dietary patterns were retained. The scree plot is provided in Supplementary material. Subsequently, the Varimax orthogonal rotation method was applied to the factor matrix to produce uncorrelated factors, facilitating simpler data interpretation. Next, to identify the important food items for each pattern, food items with rotated factor loadings >0.3 or < −0.3 were retained (14, 18). The naming and interpretation of the patterns were determined based on these retained food items. Dietary pattern scores were calculated for each participant and categorized into quartiles. The characteristics of the study population were summarized as frequencies and percentages for categorical variables and as means ± standard deviations (SD) for continuous variables. Differences in demographic and clinical characteristics between two groups were assessed based on an analysis of categorical variables using the Chi-square test, independent t-tests for normally distributed variables, and non-parametric Mann–Whitney or Wilcoxon tests for skewed variables. Binary logistic regression analysis was performed to explore the association between dietary pattern score quartiles and hypertension, using Quartile 1 (Q1) as the reference group. Demographic characteristics and comorbidities that might be associated with hypertension were included as covariates in the model. Model 1 adjusted for demographic factors such as age (per 1 year), gender (male or female), living area (urban, farming, or pastoral area), education (no schooling, primary school, middle school, or > middle school), physical activity (levels 1, 2, 3, or 4), current smoking status (smoker or non-smoker), and current drinking status (drinker or non-drinker), while Model 2 further adjusted for comorbidities, including diabetes (diabetic or non-diabetic), dyslipidemia (dyslipidemic or non-dyslipidemic), and overweight/obesity (overweight/obese or non-overweight/non-obese). The results were reported as odds ratios (OR) with 95% confidence intervals (95% CI) to quantify the strength of the association between dietary patterns and hypertension. We conducted tests for linear trend by entering the median value of each category of dietary pattern score as a continuous variable in the models. A *P*-value of <0.05 was considered statistically significant. All statistical analyses were conducted using IBM SPSS Statistics version 23.

## 3 Results

### 3.1 Participant characteristics

This study included a total of 1,262 Tibetan residents, with 38.7% from farming areas, 29.6% from pastoral areas, and 31.6% from urban areas. The study population had a mean age of 46 ± 15 years, with 36.8% being male. The prevalence rates of hypertension, diabetes, overweight/obesity, and dyslipidemia were 30.2, 5.5, 61.9, and 50.2%, respectively. Men were observed to be more likely than women to have higher BMI, SBP, and TG levels, and lower HDL-C levels. No significant differences were noted between the two genders for DBP and LDL-C. A greater proportion of men were current smokers, consumed alcohol, and were overweight or obese, while a higher proportion of women had diabetes. No significant differences were found in the prevalence of hypertension or dyslipidemia between genders (Table 1).

**Table 1:** Participant characteristics.

Variables	Total (*n* = 1,262)	Male (*n* = 487)	Female (*n* = 775)	*P*-value
Age, yrs	46.18 ± 14.57	43.58 ± 15.19	47.80 ± 13.72	<0.001^#^
BMI, kg/m^2^	25.74 ± 4.26	26.26 ± 4.25	25.41 ± 4.23	0.001^*^
SBP, mmHg	123.93 ± 21.18	125.67 ± 20.51	122.84 ± 21.53	0.021^*^
DBP, mmHg	75.66 ± 13.05	76.35 ± 13.46	75.22 ± 12.77	0.137
FBG, umol/L	4.94 ± 1.34	4.62 ± 1.18	5.13 ± 1.40	<0.001^#^
TC, mmol/L	4.75 ± 1.14	4.61 ± 1.16	4.83 ± 1.12	0.001^*^
TG, mmol/L	1.18 ± 0.70	1.23 ± 0.78	1.14 ± 0.63	0.038^*^
LDL-C, mmol/L	2.79 ± 0.92	2.73 ± 0.96	2.82 ± 0.90	0.086
HDL-C, mmol/L	1.35 ± 0.51	1.22 ± 0.32	1.43 ± 0.58	<0.001^#^
Living area, *n* (%)				
Urban area	399 (31.6)	139 (28.5)	260 (33.5)	0.007^*^
Farming area	489 (38.7)	179 (36.8)	310 (40.0)	
Pastoral area	374 (29.6)	169 (34.7)	205 (26.5)	
Education, *n* (%)				
No school	747 (59.2)	290 (59.5)	457 (59.0)	0.093
Primary school	271 (21.5)	98 (20.1)	173 (22.3)	
Middle school	130 (10.3)	44 (9.0)	86 (11.1)	
>Middle school	114 (9.0)	55 (11.3)	59 (7.6)	
Physical activity, *n* (%)				
Level 1	288 (22.8)	124 (25.5)	164 (21.2)	0.004^*^
Level 2	302 (23.9)	119 (24.4)	183 (23.6)	
Level 3	303 (24.0)	91 (18.7)	212 (27.8)	
Level 4	369 (29.2)	153 (31.4)	216 (27.9)	
Current smoking, *n* (%)	29 (2.3)	28 (5.7)	1 (0.1)	<0.001^#^
Current drinking, *n* (%)	32 (2.5)	31 (6.4)	1 (0.1)	<0.001^#^
Hypertension, *n* (%)	381 (30.2)	330 (67.8)	551 (71.1)	0.209
Diabetes mellitus, *n* (%)	69 (5.5)	11 (2.3)	58 (7.5)	<0.001^#^
Overweight/Obesity, *n* (%)	781 (61.9)	321 (65.9)	460 (59.4)	0.02^*^
Dyslipidemia, *n* (%)	634 (50.2)	250 (51.3)	384 (49.5)	0.537

SBP, systolic blood pressure; DBP, diastolic blood pressure; BMI, body mass index; FBG, fasting blood glucose; HDL-C, high-density lipoprotein cholesterol; LDL-C, low-density lipoprotein cholesterol; TC, total cholesterol; TG, triglycerides. A *P*-value < 0.05 was considered as statistically significant; *P < 0.05; ^#^*P* < 0.001.

### 3.2 Identification of major dietary patterns

Table 2 presents the factor loadings for the three major dietary patterns. Three distinct dietary patterns were identified, namely “Tsamba-red meat-tuber,” “Rice-vegetable-fruit,” and “Dairy products,” which together accounted for 25.4% of the variance in total food intake. The “Tsamba-red meat-tuber” pattern was characterized by frequent consumption of Tsamba, beef, mutton, tubers, wheat and its products, and oil. The “Rice-vegetable-fruit” pattern was characterized by frequent consumption of vegetables, rice and its products, pork, fresh fruits, mushrooms, and eggs. The “Dairy products” pattern was characterized by frequent consumption of dairy and its products.

**Table 2:** Factor loading of three major dietary patterns among subjects.

Food items	“Tsamba-red meat-tuber” pattern	“Rice-vegetable-fruit” pattern	“Dairy products” pattern
Tsampa	**0.705**	-	-
Beef and mutton	**0.623**	-	-
Tubers	**0.642**	-	-
Butter tea/mike tea	**0.531**	-	-
Wheat and its products	**0.433**	-	-
Oils	**0.399**	-	-
Vegetables	-	**0.685**	-
Rice and its products	-	**0.607**	-
Pork	-	**0.581**	-
Fruits	-	**0.403**	-
Mushrooms	-	**0.399**	-
Eggs	-	**0.328**	-
Dairy and its products	-	-	**0.679**
Whole grains	-	-	-
Beans and their products	-	-	-
Poultry	-	-	-
Animal organs	-	-	-
Aquatic products	-	-	-
Nuts	-	-	-
Pastries	-	-	-
Sweet beverages	-	-	-
Tea	-	-	-
Salt	-	-	-
Total variance explained	**14.65**	**7.47**	**5.8**

Absolute values > 0.3 or < −0.3 are shown in bold.

### 3.3 Characteristics according to dietary patterns

Table 3 presents the characteristics of participants in the highest and lowest quartiles of each dietary pattern. Individuals in the highest quartile of the “Tsamba-red meat-tuber” pattern were more likely to reside in farming and pastoral areas and to have lower education levels compared to those in the lowest quartile. Individuals in the highest quartile of the “Rice-vegetable-fruit” pattern were more likely to reside in urban and farming areas, with a higher proportion having completed middle school or higher education compared to the lowest quartile. Individuals in the highest quartile of the “Dairy products” pattern were more likely to reside in urban and pastoral areas, engage in higher levels of physical activity, and demonstrate a lower prevalence of smoking and drinking compared to those in the lowest quartile.

**Table 3:** Characteristics according to dietary patterns.

Variables	“Tsamba-red meat-tuber” pattern			“Rice-vegetable-fruit” pattern			“Dairy products” pattern		
	Q1 (*n* = 316)	Q4 (*n* = 315)	*P* value	Q1 (*n* = 315)	Q4 (*n* = 315)	*P* value	Q1 (*n* = 315)	Q4 (*n* = 315)	*P* value
Age, yrs	46.36 + 14.06	45.10 + 14.56	0.27	46.39 ± 14.76	45.75 ± 14.05	0.576	46.19 ± 15.18	46.95 ± 12.79	0.5
Male, *n* (%)	116 (36.7)	126 (40)	0.414	120 (38.1)	123 (39)	0.87	120 (38.1)	113 (35.9)	0.621
Living area, *n* (%)									
Urban area	132 (41.8)	72 (22.9)	0.001^*^	89 (28.3)	123 (39)	0.001^*^	86 (27.3)	116 (36.8)	0.001^*^
Farming area	106 (33.5)	139 (44.1)		107 (34)	116 (36.8)		144 (45.7)	88 (27.9)	
Pastoral area	78 (24.7)	104 (33.0)		119 (37.8)	76 (24.1)		85 (27)	111 (35.2)	
Education, *n* (%)									
No school	168 (53.2)	197 (62.5)	0.001^*^	174 (55.2)	179 (56.8)	0.001^*^	190 (60.3)	180 (57.1)	0.064
Primary school	67 (21.2)	75 (23.8)		73 (23.2)	72 (22.9)		67 (21.3)	60 (19)	
Middle school	31 (9.8)	27 (8.6)		47 (14.9)	21 (6.7)		40 (12.7)	38 (12.1)	
>middle school	50 (15.8)	16 (5.1)		21 (6.7)	43 (13.7)		18 (5.7)	37 (11.7)	
Physical activity, *n* (%)									
Level 1	63 (19.9)	78 (24.8)	0.453	81 (25.7)	69 (21.9)	0.6	85 (27)	54 (17.1)	0.011^*^
Level 2	84 (26.6)	72 (22.9)		80 (25.4)	78 (24.8)		74 (23.5)	68 (21.6)	
Level 3	77 (24.4)	73 (23.2)		67 (21.3)	78 (24.8)		68 (21.6)	84 (26.7)	
Level 4	92 (29.1)	92 (29.2)		87 (27.6)	90 (28.6)		88 (27.9)	109 (34.6)	
Current smoking, *n* (%)	12 (3.8)	5 (1.6)	0.138	9 (2.9)	10 (3.2)	1	14 (4.4)	4 (1.3)	0.029^*^
Current drinking, *n* (%)	12 (3.8)	7 (2.2)	0.352	11 (3.5)	13 (4.1)	0.836	20 (6.3)	3 (1)	0.001^*^

Q1, 1st quartile; Q4, 4th quartile; A *P*-value < 0.05 was considered as statistically significant; *P < 0.05; ^#^*P* < 0.001.

### 3.4 Prevalence of hypertension according to dietary patterns

Significant differences were observed in the prevalence of hypertension across quartiles of the three major dietary pattern scores. As the scores for the “Tsamba-red meat-tuber” pattern increased, the prevalence of hypertension rose from 21.5 to 41.6%. In contrast, as the scores for the “Rice-vegetable-fruit” and “Dairy products” patterns increased, the prevalence of hypertension declined from 41.9 to 21.3% and from 39.4 to 24.1%, respectively. These findings are presented in Table 4.

**Table 4:** Prevalence of hypertension and logistic regression analysis between hypertension and dietary pattern.

Dietary pattern	Prevalence of Hypertension		*P*-value	Univariate analysis	*P* for trend	Model1	*P* for trend	Model2	*P* for trend
	*n*	%							
Tsampa-red meat-tuber									
Q1	68	21.5	<0.001^#^	1 (reference)	<0.001^#^	1 (reference)	<0.001^#^	1 (reference)	<0.001^#^
Q2	80	25.4		1.24 (0.86–1.80)		1.25 (0.84–1.87)		1.27 (0.84–1.90)	
Q3	106	33.5		1.84 (1.29–2.63)		1.97 (1.34–2.91)		1.92 (1.29–2.85)	
Q4	131	41.6		2.60 (1.83–3.68)		3.09 (2.10–4.54)		3.07 (2.07–4.55)	
Rice-vegetable-fruit									
Q1	132	41.9	<0.001^#^	1 (reference)	<0.001^#^	1 (reference)	<0.001^#^	1 (reference)	<0.001^#^
Q2	95	30.1		0.60 (0.43–0.83)		0.71 (0.49–1.01)		0.69 (0.48–1.01)	
Q3	87	27.5		0.53 (0.38–0.74)		0.58 (0.40–0.83)		0.57 (0.39–0.84)	
Q4	67	21.3		0.38 (0.26–0.53)		0.47 (0.32–0.69)		0.43 (0.29–0.65)	
Dairy products									
Q1	124	39.4	<0.001^#^	1 (reference)	<0.001^#^	1 (reference)	0.003^*^	1 (reference)	0.002^*^
Q2	99	31.3		0.70 (0.51–0.98)		0.75 (0.52–1.08)		0.74 (0.51–1.09)	
Q3	82	25.9		0.54 (0.39–0.76)		0.62 (0.43–0.91)		0.58 (0.39–0.86)	
Q4	76	24.1		0.49 (0.35–0.69)		0.58 (0.40–0.85)		0.55 (0.37–0.82)	

Q1: 1st quartile; Q2: 2nd quartile; Q3: 3rd quartile; Q4: 4th quartile; *n*, number. Model 1: Adjusted for age, gender, living area, education, physical activity, current smoking, and current drinking. Model 2: Adjusted for the factors in Model 1 as well as body mass index, fasting blood glucose, high-density lipoprotein cholesterol, low-density lipoprotein cholesterol, total cholesterol, triglycerides. A *P*-value < 0.05 was considered as statistically significant; *P < 0.05; ^#^*P* < 0.001.

### 3.5 Association between dietary patterns and hypertension

Logistic regression analysis was conducted to investigate the association between the three major dietary patterns and the prevalence of hypertension (Table 4). Univariate regression analysis indicated that, compared to the lowest quartile group, participants in the highest quartile of the ‘Tsamba-red meat-tuber’ pattern had a significantly higher prevalence of hypertension, with an OR of 2.60 (95% CI: 1.83–3.68; *P* for trend <0.001). Conversely, the highest quartiles of the ‘Rice-vegetable-fruit’ and ‘Dairy products’ patterns were associated with significantly lower prevalence of hypertension, with ORs of 0.38 (95% CI: 0.26–0.53; *P* for trend <0.001) and 0.49 (95% CI: 0.34–0.69; *P* for trend <0.001), respectively. After adjusting for confounding factors, including sex, age, living area, education level, physical activity, current smoking, current alcohol consumption, BMI, FBG, TC, TG, LDL-C, HDL-C, these associations remained statistically significant. The adjusted OR for the highest quartile of the ‘Tsamba-red meat-tuber’ pattern was 3.07 (95% CI: 2.07–4.55; *P* for trend <0.001), while the adjusted ORs for the highest quartiles of the ‘Rice-vegetable-fruit’ and ‘Dairy products’ patterns were 0.43 (95% CI: 0.29–0.65; *P* for trend <0.001) and 0.55 (95% CI: 0.37–0.82; *P* for trend =0.002), respectively.

## 4 Discussion

In this study, we conducted a dietary survey using a FFQ among 1,262 Tibetan residents in farming, pastoral, and urban areas of Luhuo County in Garze Tibetan Autonomous Prefecture in Sichuan Province. Three major dietary patterns were identified among Tibetan residents: the “Tsamba-red meat-tuber,” “Rice-vegetable-fruit” and “Dairy products” patterns. The “Tsamba-red meat-tuber” pattern contributed the highest factor load (14.62%) and was considered the main dietary pattern among Tibetan residents, consistent with previous studies (18, 24). The “Rice-vegetable-fruit” pattern was similar to the main pattern of southern Chinese residents who mainly consume rice, vegetables, fruits and meat, more common in urban areas and among highly educated Tibetan residents (25). These changes may be related to urbanization in Tibetan areas, increased cultural exchange between Tibetan and Chinese cuisines, improved transportation and logistics, and greater food diversity (26, 27). Furthermore, as Tibetans originated from a nomadic ethnic group, dairy and its products remain important food sources. Our research found that Tibetans in pastoral areas, urban areas, with more physical activity and healthier lifestyles tended to prefer the “Dairy products” pattern.

This study found that the “Tsamba-red meat-tuber” pattern was a significant risk factor for hypertension among Tibetan residents. Beef and mutton, classified as red meat, are the primary food components of the “Tsamba-red meat-tuber” pattern. Both cross-sectional and longitudinal studies have demonstrated a positive association between red meat and hypertension (28). A recent umbrella review that included 43 meta-analyses found that higher consumption of red meat, particularly processed meat, was associated with an increased risk of hypertension. Further dose–response analysis revealed that an additional 100 g/day of red meat intake was positively associated with a 14% increased risk of hypertension, while consuming more than 50 g of processed meat per day was associated with a 12% increased risk of hypertension (29). Although this study did not examine the cooking methods or consumption levels of red meat, based on previous research, we suggest that red meat intake plays a significant role in the development of hypertension within the “Tsamba-red meat-tuber” pattern. Several mechanisms may explain the hypertension risk associated with red meat (28). Firstly, compared to unprocessed red meat, processed red meat—such as that which is cured, dried, fermented, or smoked—has a sodium content that is 400% higher (28). High sodium intake has been shown to be associated with elevated blood pressure and an increased risk of hypertension through mechanisms such as increasing extracellular volume, vascular resistance, and sympathetic activity, as well as worsening endothelial inflammation (30–32). Furthermore, red meat, particularly processed red meat, contains nitrite additives, with nitrite levels 50% higher than in unprocessed red meat (33). Studies have reported that each increase in the tertile of nitrate consumption is associated with a 3.1 mmHg increase in diastolic BP (34). Nitrite additives have been found to cause endothelial dysfunction, a key pathophysiological factor in the development of hypertension (35). Moreover, red meat is rich in acylcarnitines, which are metabolized by intestinal microbiota and hepatic enzymes into trimethylamine-N-oxide (TMAO). TMAO has been associated with several adverse effects, including endothelial dysfunction, atherosclerosis, oxidative stress, and vascular aging, all of which could contribute to the development of hypertension (36, 37). Additionally, the “Tsamba-red meat-tuber” pattern is characterized by a higher intake of tubers, particularly potatoes. Some studies have found inconsistent results regarding the relationship between potato intake and hypertension, with the cooking method being a factor (38–40). However, a recent meta-analysis found no association between total potato intake and hypertension, though both fried and non-fried potato consumption may increase the risk of diabetes (41). Boiled white potatoes, with a glycemic load of 21 (42), can cause postprandial hyperglycemia, which is associated with endothelial dysfunction, oxidative stress, and inflammation—factors that may negatively impact BP regulation (8). Furthermore, tsamba, a staple food in this dietary pattern, is made from naked barley. Animal studies have shown that supplementation with partly milled highland barley in high-fat diet (HFD)-fed mice significantly reduces FBG, improves oral glucose tolerance, and prevents HFD-induced gut microbiota dysbiosis (43). However, Tibetans often mix tsamba with salt and butter, which may diminish its protective effects. Therefore, the “Tsamba-red meat-tuber” pattern, characterized by high consumption of beef, mutton, and tubers, may contribute to the elevated hypertension risk observed in this population. Nevertheless, few studies have explored the dietary habits of Tibetans and their relation to hypertension. For example, Peng et al. conducted a study on Tibetan adults in the Tibetan Plateau and identified a “Pastoral pattern” similar to the “Tsamba-red meat-tuber” pattern. This pattern, which includes high consumption of red meat, tsamba, Tibetan cheese, butter tea/milk tea, and whole-fat dairy, was not associated with elevated BP (18). These findings may stem from the higher intake of whole-fat dairy in the “Pastoral pattern,” which has been found to act as a protective factor against hypertension (44), potentially offsetting the harmful effects of red meat on BP.

The Chinese traditional southern dietary pattern, characterized by a higher intake of vegetables, fruits, rice, pork, poultry, and aquatic products, has been shown to be associated with a reduced incidence of hypertension and a lower risk of future cardiovascular disease (25, 45). In our study, the “Rice-vegetable-fruit” pattern, which shares similarities with the traditional southern dietary pattern but contains relatively lower amounts of poultry and aquatic products, was observed to be associated with a lower incidence of hypertension among Tibetan residents. The observed antihypertensive effect may be attributed to the higher intake of vegetables and fruits. A recent meta-analysis of prospective studies found that a high intake of fruits and vegetables was associated with a reduced risk of hypertension, although the results for specific subtypes remain inconclusive and warrant further research (46). Several mechanisms may explain the association between vegetable and fruit consumption and lower BP. First, these foods are rich in dietary fiber, which helps regulate BP by improving vascular endothelial function, enhancing mineral absorption, reducing serum cholesterol levels, improving gastrointestinal function, and decreasing insulin resistance (47). Furthermore, fruits and vegetables are high in potassium, magnesium, vitamin C, folate, flavonoids, and carotenoids, which are thought to lower blood pressure by enhancing endothelial function, modulating baroreceptor sensitivity, and increasing antioxidant activity (48, 49). Furthermore, the “Rice-vegetable-fruit” pattern features rice as the primary staple. While a prospective cohort study conducted in China reported an inverse association between rice intake and the risk of future cardiovascular events (45), a meta-analysis found no significant association between white rice consumption and specific chronic conditions (50). The association between rice intake, hypertension, and cardiovascular events requires further investigation. Moreover, while the role of red meat in hypertension has been emphasized, the “Rice-vegetable-fruit” pattern includes higher pork intake. Possible explanations for this contradiction include: (1) the BP-lowering effects of vegetables and fruits may counteract the impact of pork; (2) studies have shown a U-shaped relationship between red meat intake and new-onset hypertension, with the risk increasing beyond a certain threshold (51). The amount of pork intake may thus influence its effect on hypertension. Additionally, inconsistent findings have been reported. A community-based nationwide study conducted in Eastern China identified a “Rice-vegetable” dietary pattern, characterized by high consumption of vegetables, rice and rice products, and aquatic products, but did not find an association with hypertension. The study suggested that the potential antihypertensive benefits of vegetable consumption could be counterbalanced by the adverse effects of polished rice, oil, and salt commonly used in stir-frying vegetables in Chinese cuisine (52). These discrepancies may arise from differences in cooking methods or the ingredients themselves, suggesting the need for further research to clarify their impact.

Our study found that the ‘Dairy products’ pattern was associated with a lower risk of hypertension among Tibetan residents. The Tibetan ethnicity, which evolved from a nomadic heritage, continues the habitual consumption of dairy products. These dairy products commonly include yogurt, whole-fat milk, cheese, and butter residue, with yogurt being the most frequently consumed item. Recent meta-analyses have shown that total dairy consumption is associated with a lower risk of hypertension, especially low-fat dairy and milk, while yogurt is more strongly linked to a reduced risk of diabetes and overweight or obesity (44). The BP-lowering effects of dairy consumption have been attributed to several components, including calcium, vitamin D, magnesium, potassium, and whey protein, which may regulate BP by enhancing insulin sensitivity, promoting renal sodium excretion, lowering intracellular calcium concentrations, and increasing nitric oxide synthesis (53–55). Additionally, yogurt, which is rich in probiotics, has been shown to lower cholesterol levels and inhibit angiotensin-converting enzyme, thereby contributing to reduced BP (56). In line with our study results, Ruan and colleagues conducted a dietary survey in Diqing of Yunnan Province, southwest China, involving Han and multi-ethnic populations, and found that a “Grassland healthy” dietary pattern, characterized by a relatively high intake of yogurt, soy products, and eggs, was associated with a lower risk of hypertension (14).

There are some limitations to this study. First, the survey sample was restricted to Luhuo County in Ganzi Tibetan Autonomous Prefecture, Sichuan Province; therefore, the research results may not be generalizable of all Tibetan populations. Second, the cross-sectional design of the study limits the ability to establish a causal relationship between dietary patterns and hypertension. Third, the findings are based on self-reported dietary information, which may be subject to recall bias. Lastly, the study did not differentiate between processed and unprocessed red meat consumption, which limits the in-depth analysis of potential mechanisms by which red meat might affect hypertension.

## 5 Conclusion

In summary, our study identified three primary dietary patterns among the Tibetan population: the “Tsamba-red meat-tuber,” the “Rice-vegetable-fruit,” and the “Dairy products” pattern. The “Tsamba-red meat-tuber” pattern is associated with a higher risk of hypertension, whereas the “Rice-vegetable-fruit” and “Dairy products” patterns are associated with a lower risk of hypertension in this population. This study provides a theoretical foundation for developing dietary strategies aimed at preventing and managing hypertension among Tibetans, with a particular focus on the Garze Tibetan Autonomous Prefecture in Sichuan Province. Future prospective studies are still needed to establish the causality between these dietary patterns and hypertension.

## Data Availability

The original contributions presented in the study are included in the article/Supplementary material, further inquiries can be directed to the corresponding author.
