# Supplementary figures and images for: Association of dietary patterns with hypertension among adults residing in Tibetan China: findings from a population-based study
Source: Front Nutr. 2025 Mar 13;12:1534915. doi: 10.3389/fnut.2025.1534915 (PMC11966421; doi:10.3389/fnut.2025.1534915)

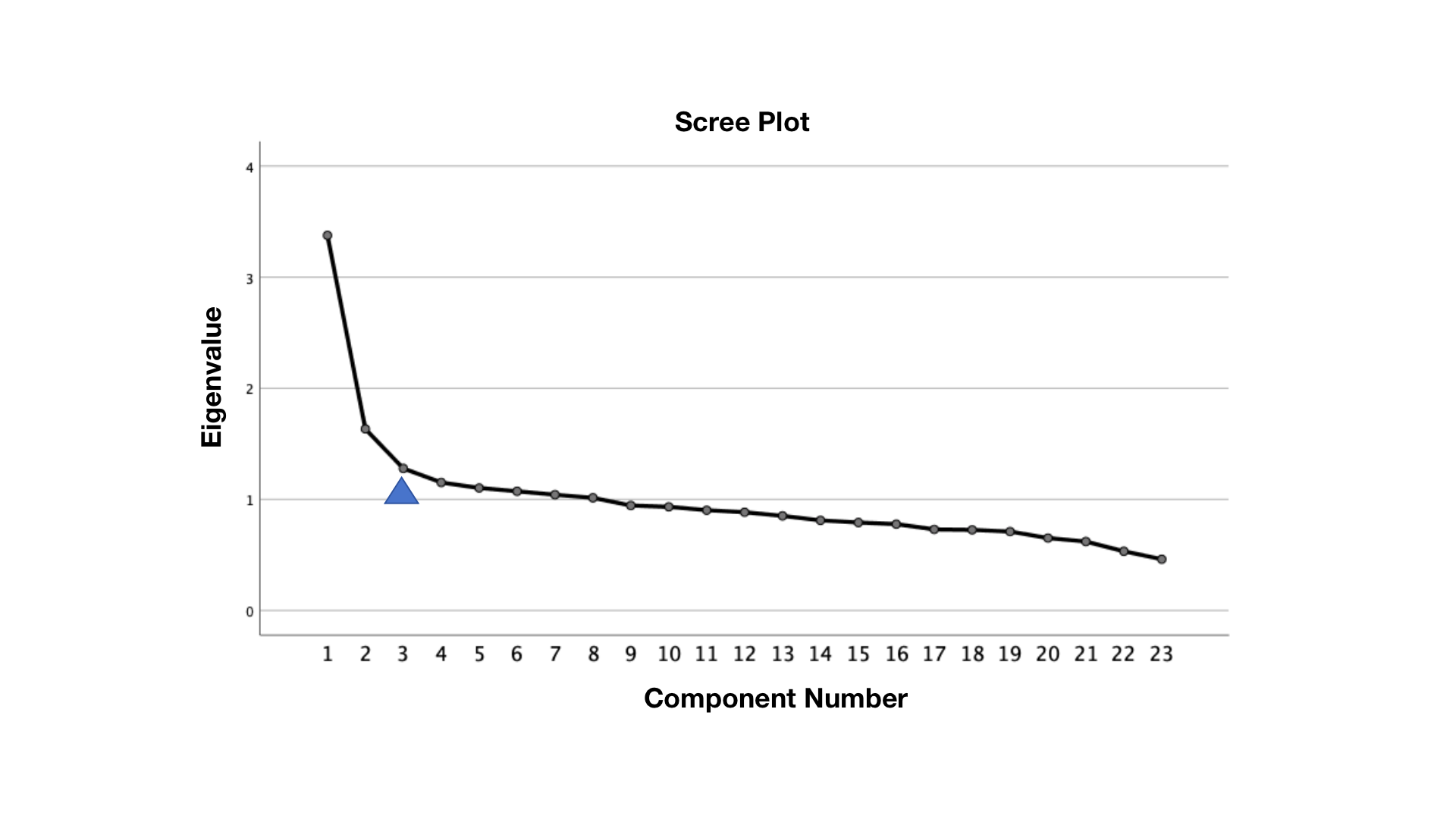

Supplement: Supplementary file 1 [file Image_1.TIF]
