# Supplementary material for: Association of dietary patterns with hypertension among adults residing in Tibetan China: findings from a population-based study
Source: Front Nutr. 2025 Mar 13;12:1534915. doi: 10.3389/fnut.2025.1534915 (PMC11966421; doi:10.3389/fnut.2025.1534915)
Supplement: Supplementary file 2 [file Table_1.DOCX]

| **Supplementary File 1. Overview of 23 Food Groups** | |
| --- | --- |
| **Food Groups** | **Food Items** |
| Rice and its products | Cooked Rice, Rice Porridge |
| Wheat and its products | Noodles, Steamed plain buns, Stuffed Steamed Buns, Dough Pieces, Pan-fried flatbread, Dumplings |
| Tsamba | Tsamba |
| Whole grains | Whole grains |
| Tubers | Potato, Sweet potato, Lotus root, Chinese yam​ |
| Beans and their products | Tofu, Dried tofu, Soy milk, Mixed legumes |
| Vegetables | Chinese cabbage, Bok choy, Spinach, Cabbage, Lettuce, Celery, Cauliflower, Rapeseed greens, Water spinach, Celtuce, Onion, Garlic chives, Garlic, Scallion, Pumpkin, Cucumber, Winter melon, Bitter melon, Eggplant, Tomato, Chili pepper, Chinese leafy greens, Luffa, Zucchini, Daikon radish, Carrot, Bean sprouts, Other legume vegetables |
| Mushrooms | Kelp, Shiitake mushroom, Wood ear mushroom, White mushroom, Enoki mushroom |
| Pork | Pork meat, Pork ribs​ |
| Beef and mutton | Beef, Yak meat, Air-dried beef, Lamb​ |
| Poultry | Chicken, Duck |
| Animal organs | Animal organs |
| Aquatic products | Freshwater fish, Shrimp |
| Eggs | Chicken egg, Duck egg |
| Dairy and its products | Milk, Yogurt, Cheese curd, Dried milk curd​ |
| Fruits | Apple, Pear, Citrus fruits, Watermelon, Banana, Grape, Peach |
| Nuts | Sunflower seeds, Walnut, Peanut​ |
| Pastries | Cake, Cookie |
| Butter tea/milk tea | Buttered Tea, Milk Tea |
| Sweet beverages | Carbonated Beverages, Fruit Juice |
| Tea | Plain Tea​ |
| Salt | Table Salt, Soy Sauce |
| Oils | Canola Oil, Salad Oil, Lard, Ghee​ |
